# The Influence of Ultra-Processed Foods on Inflammation and Metabolic Health in Pediatric Obesity: A Systematic Review with a Narrative Synthesis

**DOI:** 10.3390/nu18081186

**Published:** 2026-04-09

**Authors:** Debora Porri, Malgorzata Wasniewska, Alessandra Li Pomi, Elisa La Rosa, Giovanni Luppino, Aurora Lanzafame, Cecilia Lugarà, Roberto Coco, Francesca Franchina, Tiziana Abbate, Carla Fazio, Valentina La Malfa, Letteria Anna Morabito, Giorgia Pepe, Mariella Valenzise, Maria Francesca Messina, Domenico Corica, Tommaso Aversa

**Affiliations:** 1 Department of Human Pathology of Adulthood and Childhood, University of Messina, Via Consolare Valeria, 98124 Messina, Italy; debora.porri@unime.it (D.P.); alessandra.lipomi92@gmail.com (A.L.P.); giovilup97@gmail.com (G.L.); auroralanzafamemd@gmail.com (A.L.); cecilialug@gmail.com (C.L.); cocoroberto93@gmail.com (R.C.); francesca.franchina8@gmail.com (F.F.); tiziana.abbate93@gmail.com (T.A.); fazicarla@gmail.com (C.F.); valentinalamalfa@tiscali.it (V.L.M.); letteria.morabito@gmail.com (L.A.M.); giorgia.pepe@unime.it (G.P.); mariella.valenzise@unime.it (M.V.); francesca.messina@unime.it (M.F.M.); domenico.corica@unime.it (D.C.); tommaso.aversa@unime.it (T.A.); 2 Pediatric Unit, “G. Martino” University Hospital, 98124 Messina, Italy; elisalarosa@icloud.com

**Keywords:** ultra-processed foods, pediatric obesity, inflammation, metabolic health, diet, children, adolescents

## Abstract

The increasing prevalence of childhood obesity has been accompanied by an increase in consumption of ultra-processed foods (UPF), characterized by high energy density and low nutritional quality. Emerging evidence suggests that dietary patterns rich in UPF may contribute to low-grade systemic inflammation and early metabolic dysfunction in children and adolescents. Objective: This systematic review aimed to evaluate the association between UPF consumption and markers of inflammation and metabolic health in pediatric populations. Methods: A systematic literature search was conducted on PubMed in accordance with PRISMA guidelines. Observational and interventional studies evaluating UPF intake or diet-related inflammatory potential in children and adolescents (≤18 years) were included. Outcomes of interest included inflammatory biomarkers (e.g., C-reactive protein, interleukins, tumor necrosis factor-α) and metabolic parameters (e.g., insulin resistance, lipid profile, glucose metabolism). Results: A limited number of studies have directly assessed UPF consumption using the NOVA classification. Overall, these studies suggest a potential association between increased UPF intake and adverse metabolic outcomes, although findings on inflammatory markers remain inconsistent. A larger body of indirect evidence, including studies assessing dietary inflammatory indices and related dietary patterns, consistently supports a link between pro-inflammatory diets and increased inflammation and metabolic dysregulation in pediatric populations. Conclusions: Although direct evidence on UPF consumption remains limited, the available findings, supported by complementary indirect evidence, suggest a plausible relationship between UPF-rich diets, inflammation, and metabolic health in children and adolescents. Further well-designed studies are needed to clarify causality and improve the standardization of dietary assessment methods.

## 1. Introduction

Childhood obesity has become one of the most pressing public-health challenges worldwide, and global estimates indicate a dramatic rise in overweight and obesity among children and adolescents, with prevalence increasing across nearly all regions and socioeconomic groups [1,2,3]. Currently, approximately one in five young people has excess body weight, and projections suggest continued growth in prevalence if effective prevention and treatment strategies are not implemented [1,2].

The health consequences of pediatric obesity extend beyond increased body mass index (BMI) and include a wide spectrum of metabolic, cardiovascular, hepatic, respiratory, and psychosocial complications [4,5,6,7]. Excess adiposity in youth is strongly associated with insulin resistance, dyslipidemia, hypertension, Metabolic Dysfunction-Associated Steatotic Liver Disease (MASLD), obstructive sleep apnea, and early vascular remodeling [5,6].

A key pathophysiological component is chronic low-grade systemic inflammation, characterized by elevated circulating levels of C-reactive protein (CRP), interleukin-6 (IL-6), and tumor necrosis factor-α (TNF-α), which contribute to metabolic dysregulation and increased cardiometabolic risk [7,8,9]. Since childhood and adolescence represent critical windows of developmental plasticity, inflammatory and metabolic disturbances during this period may have long-term implications for adult morbidity.

In parallel with the rise in pediatric obesity, global dietary patterns have shifted markedly toward increased consumption of UPFs [10]. According to the NOVA classification [11], UPFs are industrial formulations composed predominantly of food-derived substances (e.g., refined starches, seed oils, sugars, protein isolates) combined with additives such as emulsifiers, colorants, flavor enhancers, thickeners, and non-nutritive sweeteners designed to enhance palatability, convenience, and shelf stability [12,13]. Their high energy density, low satiety potential, poor micronutrient content, and aggressive commercial promotion have contributed to their rapid integration into modern diets. In many high-income countries, UPFs contribute over half of total daily energy intake, and similar trends are emerging in low- and middle-income settings, particularly among children and adolescents [10,11,12,13,14,15].

Growing evidence links UPF consumption to adverse health outcomes, including weight gain, excess adiposity, metabolic syndrome, type 2 diabetes, and cardiovascular disease [15,16,17]. Importantly, the health effects of UPFs appear to extend beyond their unfavorable nutrient profile. Multiple reviews have identified additional mechanisms by which UPFs may promote obesity and metabolic dysfunction, including impaired food matrix structure, rapid nutrient absorption, disruption of appetite and reward signaling, high glycemic load, altered gut microbiota, and increased intestinal permeability [13,16,18]. Industrial processing can also generate harmful compounds—such as advanced glycation end-products (AGEs) and acrylamide—that may stimulate oxidative stress and inflammation [18,19].

Despite rapidly accumulating evidence, the extent to which UPFs influence inflammatory and metabolic outcomes specifically in children and adolescents remains insufficiently synthesized. Given the vulnerability of pediatric populations, the global rise in UPF consumption, and the recognized role of inflammation in obesity-related metabolic disease, a systematic evaluation of the current literature is urgently needed.

Therefore, this review aims to comprehensively assess observational and interventional studies examining the relationship between UPF intake, pro-inflammatory diets and markers of inflammation and metabolic health in children and adolescents, and to highlight gaps that may inform future research and public-health policy.

## 2. Methods

### 2.1. Protocol and Reporting Guidelines

This systematic review is conducted in accordance with the Preferred Reporting Items for Systematic Reviews and Meta-Analyses (PRISMA) 2020 guidelines [20]. The PRISMA flow diagram is used to illustrate the study selection process.

### 2.2. Eligibility Criteria

Studies were selected according to the PICOS framework.

Population: Children and adolescents (≤18 years), including both the general pediatric population and those with overweight or obesity, provided that metabolic or obesity-related outcomes were reported.

Exposure: Dietary exposures reflecting consumption of ultra-processed foods (UPF). This included: (i) direct assessment of UPF intake, defined according to the NOVA classification or equivalent descriptions for industrially formulated foods; and (ii) indirect measures capturing dietary patterns or characteristics strongly associated with UPF-rich diets, such as dietary inflammatory indices (e.g., Dietary Inflammatory Index) or empirically derived dietary patterns indicative of pro-inflammatory or Western-style diets. This broader exposure definition was predefined to account for the limited number of pediatric studies directly quantifying UPF consumption and was systematically applied during study selection.

Comparisons: Lower consumption of ultra-processed foods (UPF), healthier dietary patterns, or comparisons across categories of inflammatory potential of foods.

Outcomes: Markers of inflammation (e.g., C-reactive protein, interleukins, tumor necrosis factor-α, adipokines) and/or metabolic health (e.g., insulin resistance, glucose metabolism, lipid profile, components of metabolic syndrome).

Study design: Original observational studies (cross-sectional, cohort, case–control) and interventional studies.

### 2.3. Exclusion Criteria

Exclusion criteria were defined as follows:

(i) Studies conducted exclusively on adult populations;
(ii) Studies that did not assess dietary exposure relevant to the consumption of ultra-processed foods, either directly (e.g., NOVA classification) or indirectly through validated proxy measures (e.g., dietary inflammatory indices or dietary patterns associated with diets high in ultra-processed foods);
(iii) Studies that focused exclusively on single nutrients or isolated food components (e.g., sugars or fats) without considering overall dietary patterns;
(iv) Non-original research, including reviews, meta-analyses, editorials, letters, conference abstracts, and animal studies.

These criteria were applied consistently to ensure consistency between the defined exposure framework and the studies included in the review.

### 2.4. Information Sources and Search Strategy

A systematic literature search was conducted in PubMed from September 2025 to January 2026. The search strategy combined controlled vocabulary and free-text terms related to ultra-processed foods, pediatric obesity, inflammation, and metabolic health. Reference lists of included articles were also screened to identify additional relevant studies. Eligibility criteria included articles published in the last 10 years.

All records identified through the search were imported into a reference management software, and duplicates were removed. Two reviewers independently screened titles and abstracts to identify potentially eligible studies. Full-text articles were then retrieved and assessed for eligibility. Disagreements were resolved through discussion or consultation with a third reviewer when necessary.

Data were independently extracted by two reviewers using a standardized data extraction form. Extracted information included: author and year of publication; study design and country; sample size and participant characteristics; definition and assessment of ultra-processed food consumption; inflammatory and metabolic outcomes assessed; main findings.

### 2.5. Data Synthesis

Given the heterogeneity in study designs, exposure definitions, and outcome measures, a quantitative meta-analysis was not feasible. Therefore, a qualitative narrative synthesis was performed. The evidence was categorized into:

(i) direct evidence, including studies that explicitly assessed UPF consumption according to the NOVA classification; and
(ii) indirect evidence, including studies evaluating dietary inflammatory potential or dietary patterns associated with UPF-rich diets.

This approach was adopted to provide a comprehensive overview of both direct associations and underlying mechanistic pathways linking diet, inflammation, and metabolic health in pediatric populations.

## 3. Synthesis of Results

### 3.1. Overview

**Figure 1:**
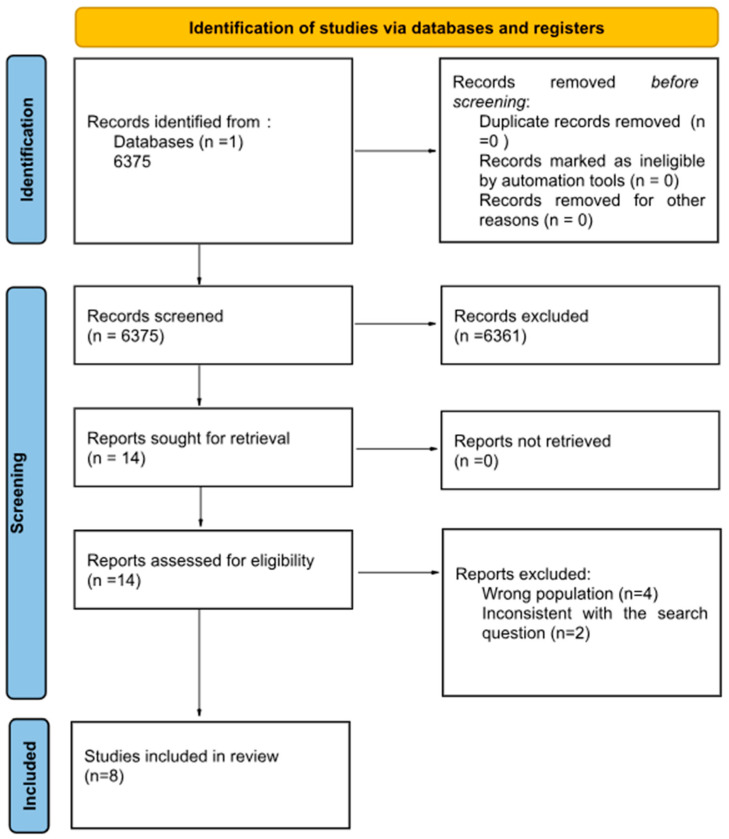
Prisma Flow Diagram.

A flowchart summarizing the study selection process is presented in Figure 1. The electronic searches return 6375 records. Fourteen studies are retained after examining the titles and abstracts, while six studies are further excluded after reading the full texts. Of the six excluded records, two referred to the wrong population (children with other conditions), while the other four studies were inconsistent with the search question. Only eight eligible studies are included in this systematic review. A flowchart summarizing the study selection procedure is presented in Figure 1.

### 3.2. Results

#### Study Characteristics

Given the heterogeneity of study designs, exposure definitions, and outcome measures, a qualitative narrative synthesis is performed.

Only two studies [21,22] directly assessed ultra-processed food intake; the remaining evidence [23,24,25,26,27,28] provides indirect, hypothesis-generating evidence that contributes to the interpretation of potential mechanisms rather than directly addressing the primary exposure of interest. Given the limited number of pediatric studies directly assessing UPFs consumption in relation to inflammatory biomarkers, the present review also considered studies examining diet-related inflammatory potential or dietary patterns known to be strongly associated with UPF-rich dietary profiles. This approach was adopted to contextualize the potential mechanistic relationship between diet quality, inflammation, and metabolic health in pediatric populations.

Mete et al. [21] conducted a cross-sectional study in children aged 5–17 years, examining the association between UPF intake and low-grade systemic inflammation assessed through a composite inflammatory score (INFLA-score). Dietary intake is evaluated using repeated 24 h recalls classified according to the NOVA system. The study found that a higher proportion of daily energy intake from UPFs is significantly associated with increased inflammatory burden, independent of obesity status. These findings suggest that UPF consumption may contribute to early inflammatory processes even before overt metabolic dysfunction becomes evident.

Similarly, Martins et al. [22] analyzed data from a population-based cross-sectional study of Brazilian adolescents aged 17–18 years. UPF intake is quantified using dietary recalls and NOVA classification, while inflammation is assessed through circulating biomarkers, including C-reactive protein, interleukin-8, leptin, and adiponectin. Adolescents with higher UPF consumption exhibited significantly elevated levels of pro-inflammatory markers, particularly IL-8 and leptin, after adjustment for potential confounders. These results indicate that diets high in UPFs are associated with a pro-inflammatory profile during adolescence.

Together, these two studies provide consistent evidence linking higher consumption of UPFs with systemic inflammation in pediatric populations. Although limited by their cross-sectional design and small number, the findings support the hypothesis that UPFs intake may play a role in early inflammatory pathways relevant to pediatric obesity and future metabolic health.

The study characteristics are summarized in Table 1.

Five additional studies [23,24,25,26,27,28] are included even though they do not directly meet the predefined exposure definition for UPFs, as they examine the association between diet-related inflammatory potential and metabolic health in pediatric populations (Table 2). These studies were not considered as direct evidence addressing the primary research question; rather, they were included to provide mechanistic and contextual insight into the relationship between diet-related inflammatory potential and metabolic health in pediatric populations. UPFs represent a major component of Western-style and pro-inflammatory dietary patterns, which are typically characterized by high intakes of refined carbohydrates, added sugars, saturated fats, and low dietary fiber. Therefore, studies evaluating the inflammatory potential of the overall diet—such as those using the Dietary Inflammatory Index (DII) or dietary pattern analyses—offer complementary evidence relevant to the research question. Although these studies do not directly quantify UPF intake according to the NOVA classification, they help contextualize the broader dietary environment and provide mechanistic insight into the inflammatory pathways through which UPF-rich diets may influence metabolic health in pediatric populations. These studies reflect a composite measure derived from previous associations between dietary components and inflammatory biomarkers. Therefore, their findings should be interpreted as indicative of the inflammatory potential of the diet, rather than as independent confirmation of the inflammatory effects of ultra-processed foods.

Latorre-Millán et al. [23], using data from the Genobox cohort, assessed dietary patterns in children and adolescents and reported associations between Western-style dietary patterns and increased adiposity, adverse lipid profiles, and cardiometabolic risk markers. Although UPF intake was not explicitly quantified, the identified dietary patterns were characterized by foods typically associated with lower nutritional quality, which may conceptually overlap with UPF-rich diets. Behrooz et al. [24] assessed adherence to the Dietary Approaches to Stop Hypertension (DASH) diet in adolescents with obesity and observed inverse associations with the prevalence of metabolic syndrome and circulating inflammatory biomarkers. This study provides comparative evidence on the potential anti-inflammatory effects of high-quality dietary patterns, serving as a contrast to dietary exposures commonly associated with higher UPF consumption. Additionally, three studies investigated the inflammatory potential of the diet using the Dietary Inflammatory Index (DII), validated by Cavicchia et al. [29], with the aim of establishing the relationship between dietary constituents and high-sensitivity C-reactive protein (hs-CRP) values. The DII is a scoring algorithm designed to evaluate an individual’s diet based on its potential to induce inflammation focused on six inflammatory markers: interleukin IL-1b, IL-4, IL-6, IL-10, TNF-α, and CRP [30]. This nutritional tool helps assess appropriate dietary goals for individuals, potentially preventing the risk of inflammation. In a nationally representative U.S. sample, Zhang et al. [25] reported that higher DII scores are associated with increased odds of overweight and obesity among children and adolescents, highlighting a population-level link between pro-inflammatory diets and excess adiposity. These findings suggest that dietary inflammatory potential may contribute to the early development of obesity, a key driver of cardiometabolic risk in pediatric populations. Beyond adiposity, evidence from Brazilian schoolchildren indicates that pro-inflammatory diets are also associated with distinct metabolic and cardiovascular risk markers. In a cross-sectional study by Suhett et al. [26], higher DII scores were independently associated with increased atherogenic risk, as reflected by unfavorable lipid ratios, even in a general pediatric population not selected for obesity or ultra-processed food intake. This finding underscores the relevance of dietary inflammatory potential for early cardiovascular risk, independent of overt adiposity. In a separate but complementary analysis of the same cohort [27], higher DII scores are associated with increased leptin concentrations and reduced adiponectin levels, indicating a shift toward a pro-inflammatory adipokine profile. Given the central role of adipokines in regulating inflammation, insulin sensitivity, and energy homeostasis, these results suggest that diet-induced inflammation may influence metabolic health through adipose tissue dysfunction from an early age. Taken together, these studies demonstrate that diets with higher inflammatory potential are associated not only with overweight and obesity but also with early alterations in lipid metabolism and adipokine signaling, which may precede and contribute to the development of cardiometabolic disease later in life. Importantly, these associations were observed in general pediatric populations and independently of direct measures of ultra-processed food consumption, supporting the hypothesis that dietary inflammatory potential represents a critical mechanistic pathway linking overall diet quality to metabolic health during childhood. Finally, Morandi et al. [28] conducted an experimental meal challenge study demonstrating that a high-fat meal induced acute increases in systemic inflammatory markers and impaired glucose homeostasis in children and adolescents with obesity. Although this study did not assess habitual dietary intake or UPFs exposure, it supports the biological plausibility of diet-induced inflammatory responses as a mechanistic pathway linking dietary composition to metabolic dysregulation.

Overall, while these studies do not directly address UPFs consumption as defined by the NOVA classification, they provide consistent indirect evidence supporting the role of diet-related inflammation in pediatric obesity and metabolic health. Their inclusion aligns with PRISMA guidance for transparent reporting of heterogeneous evidence contributing to the interpretation of the review findings.

## 4. Discussion

### 4.1. Dietary Inflammatory Potential as a Mechanistic Link Between Diet Quality and Metabolic Health

In addition to the limited direct evidence assessing UPFs consumption, the studies included in this review provide complementary, indirect insights into the potential role of diet-related inflammatory processes in pediatric metabolic health. In particular, several studies [25,26,27,28] evaluated dietary inflammatory potential using indices such as the Dietary Inflammatory Index (DII) or examined dietary patterns known to influence inflammatory pathways. While these approaches do not directly quantify UPFs intake, they offer contextually relevant and hypothesis-generating evidence that may help to elucidate the broader relationship between diet quality, inflammation, and obesity-related outcomes in pediatric populations. However, these findings should be interpreted with caution, as such indices are derived from prior associations with inflammatory biomarkers and therefore do not represent independent measures of dietary-induced inflammation.

In the included studies, higher DII scores are associated with adverse cardiometabolic profiles and inflammatory alterations. In Brazilian schoolchildren, diets with higher inflammatory potential were linked to increased atherogenic risk and unfavorable lipid ratios, as well as a pro-inflammatory adipokine profile characterized by higher leptin and lower adiponectin levels [26,27]. Similarly, analysis of US children and adolescents from the NHANES cohort showed that higher DII scores are significantly associated with increased odds of overweight and obesity [25], supporting a population-level association between dietary inflammation and adiposity.

These findings are consistent with previous pediatric studies demonstrating that pro-inflammatory dietary patterns are associated with increased systemic inflammation and metabolic risk. For example, Shivappa et al. [30] originally established DII as a predictor of inflammatory biomarkers, including C-reactive protein and interleukin-6, laying the foundation for its application in diverse populations, including children.

Subsequent work by Ruiz-Canela et al. [31] showed that higher DII scores are associated with insulin resistance and dyslipidemia, outcomes highly relevant to pediatric obesity.

Furthermore, evidence from dietary pattern analyses supports the idea that inflammatory potential reflects broader diet quality rather than isolated nutrients. In the Genobox cohort, Western dietary patterns are associated with increased fat mass and adverse cardiometabolic markers, whereas healthier patterns showed protective associations [23], such as reduced adiposity, improved lipid profiles (e.g., lower triglycerides and higher HDL cholesterol), and better glucose homeostasis. This is consistent with findings by Ambrosini et al. [32], who demonstrated that Western-style diets rich in processed foods are associated with higher concentrations of inflammatory markers in younger populations.

Importantly, dietary patterns with high inflammatory potential share several structural features with diets rich in UPFs, including excessive intakes of refined carbohydrates, saturated fats, and low fiber density. Experimental and observational evidence suggests that these dietary features promote chronic low-grade inflammation through mechanisms involving oxidative stress, adipose tissue dysfunction, and altered insulin signaling [33,34,35]. For example, Calder et al. [36] highlighted the role of macronutrient quality and food processing in modulating inflammatory pathways.

Taken together, these data support the interpretation that the dietary inflammatory index and related pattern-based approaches capture key mechanistic pathways through which poor diet quality—often characterized by high ultra-processed food consumption—may contribute to inflammation and metabolic dysregulation in pediatric obesity.

### 4.2. Protective Dietary Patterns and Anti-Inflammatory Effects in Pediatric Obesity

In contrast to pro-inflammatory dietary patterns, several studies included in this review highlight the potential protective role of anti-inflammatory dietary models in mitigating inflammation and metabolic risk among children and adolescents with obesity.

These findings reinforce the importance of dietary quality as a modifiable determinant of immune-metabolic health early in life. Among the included studies, greater adherence to the DASH dietary pattern was inversely associated with metabolic syndrome prevalence and circulating inflammatory biomarkers in adolescents with obesity [24]. Similarly, healthier dietary patterns identified in the Genobox cohort are associated with more favorable body composition and cardiometabolic profiles [23].

These results are consistent with findings from Suhett et al. [26,27], who observed that lower dietary inflammatory potential was associated with a more favorable adipokine profile, including higher adiponectin and lower leptin concentrations, suggesting improved adipose tissue function. These observations align with a broader pediatric literature indicating that anti-inflammatory dietary patterns—such as the Mediterranean diet—are associated with lower levels of systemic inflammation. These observations align with a broader pediatric literature indicating that anti-inflammatory dietary patterns—such as the Mediterranean diet—are associated with lower levels of systemic inflammation. For example, Sureda et al. [37] reported inverse associations between Mediterranean diet adherence and CRP and IL-6 concentrations. A systematic review by Bujtor and colleagues [38] demonstrated that dietary patterns and food groups rich in fruits, vegetables, and whole grains are associated with lower levels of pro-inflammatory biomarkers, including CRP, IL-6, and TNF-α, compared with Western dietary patterns, which are typically linked to higher inflammatory burden and adverse metabolic profiles. In addition, growing evidence suggests that dietary patterns modulate gut microbiota composition, which plays a critical role in immune and metabolic homeostasis. For instance, De Filippo et al. [39] provided early evidence that plant-rich diets promote a microbiota profile associated with reduced inflammatory levels, another important aspect.

In summary, these findings suggest that promoting anti-inflammatory dietary patterns may represent an effective strategy to counterbalance the adverse effects of pro-inflammatory during childhood.

Rather than focusing exclusively on the reduction of individual food components, interventions aimed at improving overall dietary quality may yield broader and more sustainable benefits for inflammation control and metabolic health in pediatric obesity.

## 5. Limitations

Several limitations of this systematic review must be acknowledged and carefully considered when interpreting the results. First, the limited number of studies and their heterogeneity in terms of population characteristics and study design reduce the generalizability of the findings.

Most of the included studies adopted a cross-sectional design, which inherently limits the ability to infer causal relationships between dietary exposures, inflammation, and metabolic outcomes. Although consistent associations were observed between pro-inflammatory dietary patterns, consumption of growth factors (UPFs), and adverse metabolic markers, the temporal direction of these relationships cannot be established. Therefore, it is unclear whether pro-inflammatory diets contribute to the development of obesity-related inflammation or whether existing metabolic alterations influence dietary behaviors.

It is also important to consider that there was substantial heterogeneity in dietary assessment methods across studies, including 24 h dietary recalls, dietary records, and derived dietary indices such as the DII. These methods are subject to recall bias, reporting bias, and day-to-day variability, which can be particularly pronounced in pediatric populations. Furthermore, differences in dietary assessment tools may limit the comparability of results across studies and contribute to variability in effect estimates. Specifically, considering the DII, because it is derived from previous associations between dietary components and inflammatory biomarkers, findings based on this index should be interpreted as a reflection of the inflammatory potential of the diet rather than as independent evidence of causality.

Furthermore, although two studies directly assessed UPFs consumption using the NOVA classification, the remaining studies relied on indirect measures of diet quality, eating patterns, or inflammatory indices. While these approaches capture broader dietary characteristics relevant to inflammation, they do not allow for precise quantification of ultra-processed food intake. Consequently, the contribution of ultra-processed foods to dietary inflammatory potential cannot be fully dissociated from other aspects of overall diet quality. Another important limitation concerns the variability of inflammatory and metabolic outcomes assessed across studies. Biomarkers ranged from systemic inflammatory markers (e.g., C-reactive protein, cytokines) to adipokines, lipid ratios, and indices of insulin resistance. This heterogeneity has impeded quantitative synthesis and limits the ability to draw conclusions about specific inflammatory pathways consistently influenced by dietary exposure. Furthermore, most of the studies included in this review are conducted in specific geographic and socioeconomic settings, primarily in high- and middle-income countries, which may limit the generalizability of the findings to other pediatric populations with different dietary patterns, food environments, and cultural determinants of diet. Furthermore, several studies did not account for potential confounding factors such as physical activity, pubertal status, socioeconomic status, and parental education, which are known to influence both dietary habits and metabolic health.

## 6. Conclusions, Clinical Implications and Future Directions

In summary, available evidence supports a consistent association between high UPFs consumption and a pro-inflammatory and metabolically unfavorable profile in children and adolescents with overweight or obesity. Highly processed diets appear to contribute to low-grade systemic inflammation, adipokine imbalance, impaired glucose homeostasis, and adverse lipid profiles. These findings reinforce the concept that the health impact of UPFs extends beyond its macronutrient composition and involves broader alterations in diet quality and inflammatory potential.

From a clinical perspective, assessing dietary patterns in pediatric obesity should go beyond caloric intake and macronutrient distribution to include the degree of food processing, from a qualitative perspective. Identifying excessive UPFs consumption may help identify children at higher risk for inflammatory and metabolic dysregulation. Nutrition interventions should prioritize reducing UPFs and promoting minimally processed, nutrient-dense foods within comprehensive, family-centered, and multidisciplinary management programs. Targeting overall dietary quality may be a more sustainable and physiologically meaningful strategy than focusing solely on individual nutrients. These findings could also inform policy actions, highlighting the need for regulatory and educational strategies aimed at reducing the availability and consumption of ultra-processed foods among children and adolescents, for example also through educational institutions.

Future research should prioritize well-designed longitudinal and interventional studies to clarify the causal relationships between ultra-processed food intake, inflammation, and metabolic outcomes in pediatric populations. The adoption of standardized methods for defining and quantifying UPFs consumption is essential to enhance comparability and reproducibility across studies. In addition, further investigation is needed to elucidate the underlying biological mechanisms, including alterations in gut microbiota, oxidative stress pathways, and adipose tissue dysfunction, as well as the role of socioeconomic and environmental determinants. A deeper understanding of critical developmental windows may help identify periods of increased vulnerability and inform the timing of targeted preventive strategies.

Overall, while direct evidence specifically linking UPFs consumption to inflammatory and metabolic outcomes in pediatric populations remains limited, the consistency of findings from indirect evidence—particularly studies assessing dietary inflammatory potential and related dietary patterns—supports a plausible and biologically coherent relationship between UPF-rich diets, low-grade inflammation, and early metabolic dysregulation.

## Figures and Tables

**Table 1:** Characteristics of studies evaluating ultra-processed food consumption and inflammation in pediatric populations.

Study (Year)	Country	Study Design	Population (Age)	Sample Size (*n*)	Dietary Assessment	UPFs Classification	Inflammatory Markers	Main Findings
Mete et al. (2024) [21]	Turkey	Cross-sectional	Children (5–17 years)	50	Three 24 h dietary recalls	NOVA	Composite INFLA-score (including CRP, leukocytes, platelets)	Higher percentage of energy intake from UPF is significantly associated with increased low-grade inflammation, independent of obesity status
Martins et al. (2022) [22]	Brazil	Cross-sectional, population-based	Adolescents (17–18 years)	391	Two 24 h dietary recalls	NOVA	CRP, IL-6, IL-8, TNF-α, leptin, adiponectin	High UPF consumption (>30% of total energy) is associated with higher IL-8, leptin, and CRP levels, indicating a pro-inflammatory profile

**Table 2:** Characteristics of studies evaluating dietary patterns, dietary inflammatory potential, and inflammation-related metabolic outcomes in pediatric populations. Acronyms: Dietary Approaches to Stop Hypertension (DASH diet); C-Reactive Protein (CRP); Interleukin 6 (IL-6); Tumor Necrosis Factor (TNF); Dietary Inflammatory Index (DII).

Study (Year)	Country	Study Design	Population (Age)	Sample Size	Dietary Exposure/Index	Inflammatory/Metabolic Outcomes	Main Findings
Latorre-Millán et al. (2020) [23]—Genobox Cohort	Spain	Cross-sectional (cohort-based)	Children and adolescents (5–18 years)	594	Dietary patterns derived from dietary records	Body composition, lipid profile, insulin resistance markers	Western dietary patterns are associated with higher fat mass, adverse lipid profile, and increased cardiometabolic risk.
Behrooz et al. (2025) [24]	Iran	Cross-sectional	Adolescents with obesity (12–18 years)	203	DASH diet adherence score	CRP, IL-6, TNF-α, metabolic syndrome components	Greater adherence to the DASH diet was inversely associated with metabolic syndrome prevalence and lower levels of inflammatory biomarkers
Zhang et al. (2024) [25]—NHANES	United States	Cross-sectional (nationally representative)	Children and adolescents (6–19 years)	>6000	Dietary Inflammatory Index (DII)	Obesity status, BMI z-score	Higher DII scores (more pro-inflammatory diets) are significantly associated with increased odds of overweight and obesity
Suhett et al. (2021) [26]	Brazil	Cross-sectional, school-based	Schoolchildren (8–12 years)	409	Dietary Inflammatory Index (DII)	Lipid ratios, atherogenic risk indices	Pro-inflammatory diets (higher DII) are associated with increased atherogenic risk, including unfavorable lipid ratios
Suhett et al. (2021) [27]	Brazil	Cross-sectional, school-based	Schoolchildren (8–12 years)	409	Dietary Inflammatory Index (DII)	Leptin, adiponectin	Higher DII scores are associated with increased leptin and reduced adiponectin levels, indicating a pro-inflammatory adipokine profile
Morandi et al. (2017) [28]	Italy	Experimental (meal challenge study)	Children and adolescents with obesity (7–18 years)	30	High-fat test meal	CRP, IL-6, glucose, insulin	A single high-fat meal induced acute systemic inflammation and impaired glucose homeostasis, highlighting postprandial inflammatory responses in pediatric obesity

## Data Availability

No new data were created or analyzed in this study.
